# Appraising the HIV prevention cascade methodology to improve HIV prevention targets: Lessons learned from a general population pilot study in east Zimbabwe

**DOI:** 10.1371/journal.pgph.0005336

**Published:** 2026-02-10

**Authors:** Louisa R. Moorhouse, Jeffrey W. Imai-Eaton, Rufuworkuda Maswera, Blessing Tsenesa, Phyllis Magoge-Mandizvidza, Brian Moyo, Owen Mugurungi, Constance Nyamukapa, Timothy B. Hallett, Simon Gregson

**Affiliations:** 1 MRC Centre for Global Infectious Disease Analysis, School of Public Health, Imperial College London, London, United Kingdom; 2 Center for Communicable Disease Dynamics, Department of Epidemiology, Harvard T.H. Chan School of Public Health, Boston, Massachusetts, United States of America; 3 Manicaland Centre for Public Health Research, Biomedical and Research Training Institute, Harare, Zimbabwe; 4 AIDS & TB Unit, Zimbabwe Ministry of Health and Child Care, Harare, Zimbabwe; Walter Sisulu University, SOUTH AFRICA

## Abstract

Multiple HIV Prevention Cascades (HPC) formulations have been proposed to assist advocacy, monitoring of HIV prevention implementation and research to identify ways to increase use of HIV prevention methods. Schaefer and colleagues proposed a unifying formulation suitable for widespread use across different populations which could be used for routine monitoring or advocacy. Robust methods for defining and interpreting this HPC formulation using real world data are necessary to aid promotion and utilisation of this framework to address necessary gaps in primary HIV prevention method use. We used 2018–19 data from the Manicaland Pilot HIV Prevention Cascades Study in eastern Zimbabwe to validate the HPC framework for pre-exposure prophylaxis (PrEP), voluntary medical male circumcision (VMMC), female and male condoms, and combination prevention. Validation involved: (1) testing feasibility of populating the HPC; (2) comparing simple vs. complex HPC measures using 2-sample proportion tests; and (3) using logistic regression to assess whether HPC cascade bars predicted prevention use and whether sub-bars explained loss from the HPC. It was possible to populate the HPC for individual and combined prevention methods using pilot survey data. Most steps were associated with prevention method usage outcomes, except for VMMC. There were significant overlaps between individuals reporting positive responses for the main motivation bar and those citing barriers to motivation. To refine the HPC’s access bar definition, it is suggested to consider individuals who report access barriers. While the HPC framework identifies barriers to individual prevention methods, challenges arise in identifying those for combined prevention. Our study successfully utilised questionnaires from the Manicaland HPC pilot survey to measure the HPC for individual and combined prevention methods. In conclusion, it is feasible to populate this framework using general population survey data and designated questionnaire modules. We propose a final formulation of the HPC, and questionnaire modules and methods to create it. With proper promotion, such as through measurement within national to local population surveys, the HPC framework can allow comparison of prevention method use, identification of intervention targets and enhance prevention services, aiding in the crucial reduction of HIV incidence.

## Introduction

It has been globally recognised that there is not a standalone approach to HIV prevention that can end the HIV epidemic and instead the focus of global HIV prevention efforts now lies on combination HIV prevention [1–5]. Combination prevention approaches centre around using context specific and evidence-based HIV prevention interventions which are adapted to specific populations and, as UNAIDS has recommended, “knowing your epidemic” [6,7]. This tailored approach to identifying and promoting HIV prevention needs and efforts requires identification of the key drivers of the epidemic within the population or context of interest, subsequently identifying the populations at highest risk of HIV acquisition and considering the most suitable interventions and prevention methods for each population [7]. The UNAIDS HIV Prevention 2025 Roadmap emphasises prioritising combination HIV prevention through comprehensive packages of HIV prevention services and sets ambitious targets for combination HIV prevention with the aim of 95% of people at risk of HIV using “appropriate, prioritised, effective combination prevention” [8].

Very few countries have successfully applied a combination HIV prevention approach including behavioural, biomedical and structural components relevant to context and populations [3]. UNAIDS note how combination prevention for young people in high HIV prevalence settings should consist of condom and behaviour change promotion, comprehensive sexuality education as well as access to services without economic or structural barriers such as cost or parental consent laws [3]. Interventions aim to increase combination prevention and include social protection, addressing community norms, reducing risk of sexual partners through testing and VMMC, parenting and caregiver programmes and improving access to sexual and reproductive health care services [9,10]. However, as Hargreaves et al discuss, this approach to combination lacked a framework to guide measurement of such multilevel factors and then use these insights to guide programme design integrating such multilevel factors, as opposed to considering them in isolation [11,12].

As noted by the UNAIDS HIV Prevention Gap Report in 2018, the rate at which new HIV infections are decreasing has declined which threatens the good progress made towards ending the HIV/AIDS epidemic. Scaling up prevention method use to prevention new HIV infections is crucial [3]. Treatment as Prevention (TasP) is insufficient alone to reduce HIV incidence rates to targeted levels [3]. Understanding multilevel components contributing to lower than anticipated prevention method use, including demand-side, supply-side and structural barriers, is crucial to reaching global targets of HIV incidence reduction. Resources are required to allow measurement of these multilevel barriers at both national and subnational levels, as well as facilitating ongoing monitoring and cross-country comparison of prevention method use.

The 2021 Political Declaration notes that condom use is declining and commits to increasing “evidence-based enabling measures for proven HIV combination prevention, including condom promotion and distribution” [5]. Additionally, a crucial point is made that while further integration of HIV prevention efforts into HIV and sexual and reproductive health programmes is very important, this, on its own, neglects those who are not engaged within these systems [13].

Following the success of the HIV Treatment Cascade framework to compare and evaluate national and sub-national HIV treatment programmes [14–16], multiple models of HIV prevention cascades (HPC) have been proposed to monitor progress of HIV prevention implementation from national to local levels, identify ways to increase use of HIV prevention methods, allow comparison across HIV prevention programmes, and ultimately to reduce HIV incidence [11,17–23].

The HIV prevention cascade (HPC) framework has been proposed to serve several different purposes including:

1) Advocacy for resources and action
2) High level monitoring of progress towards (as yet un-standardised) targets
3) Local level monitoring of progress in implementing primary prevention methods
4) Research to identify ways to increase effective use (or uptake and adherence) of HIV prevention methods

The aim of the HPC model is to provide a practical framework that indicates where HIV prevention activities need to be strengthened by describing the steps required for HIV prevention to be effectively used by an individual, and identifies barriers to the individual transitioning through each of these steps [17,18,11]. Successful application of the HPC must assist decision makers in identifying intervention uptake targets that result in improved effective use of HIV prevention [18,24].

Garnett et al proposed two general models of an HPC which were applied to data from the Manicaland cohort study – a long-running general population survey conducted in eastern Zimbabwe to investigate trends and determinants of HIV infection, prevention, and treatment within the general population [17]. The first of these models takes the view of a health care provider, i.e., provider-centric and the other takes the view of an individual potentially engaging with an HIV prevention method, i.e., user-centric [17]. These were the first of the proposed HPC models which were more generic rather than being developed for a specific population (such as men who have sex with men (MSM)) or prevention method (such as pre-exposure prophylaxis (PrEP)). Further work by Hargreaves and colleagues then modified and built on the HPC formulations proposed by Garnett et al [17]. Firstly, they identified three domains of the cascade that interventions could be related to: demand side interventions which aim to increase motivation, supply side interventions which improve availability, and adherence interventions to improve uptake and use of HIV prevention. Secondly, they incorporated known barriers in each of these domains drawn from social cognitive theoretical frameworks and the wider literature and linked these to the types of interventions most likely to reduce these barriers [11]. i.e., in effect, a theoretical framework. However, such theoretical frameworks were not designed for use in routine programme monitoring and advocacy and there was a further need for a unifying framework which could be used for such work.

Following a consultation in Harare on earlier HPC formulations, Schaefer *et al.* and the London Working Group on HIV Prevention Cascades (LWG) proposed a unifying cascade framework (the Harare HPC) for monitoring and evaluation of prevention programmes, which drew heavily on the earlier work by Hargreaves and Garnett [18]. It sets out the key steps, and subsequent gaps between these key steps, that need to be addressed to achieve effective prevention method use. The framework can be applied to multiple primary prevention methods and populations [18,11,17]. Schaefer *et al.* highlight that a successful cascade framework must be sufficiently generic to adapt across prevention methods and populations, and be efficient and practical to populate with real world data [18,25]. The resulting Harare HPC was proposed to be measured in population surveys following a user centric approach, applied to multiple populations and to be used in two parts. A simple framework was proposed consisting of the main bars of the cascade (motivation, access, effective use). Additionally, an extended version of the simple framework was proposed which considers explanatory barriers to each of the gaps in the HPC (lack of motivation, lack of access, lack of capacity to effectively use) [25]. It has been suggested in the literature that two different cascade formulations could be required—one simple model which populates the core steps and highlights gaps in the cascade to evaluate prevention programmes and one detailed model which includes explanatory factors for the gaps observed in the cascades [26]. This is similar to the approach suggested by Schaefer *et al.* whereby the core steps of the cascade framework are populated initially and then, if data are available, explanatory sub-bars are populated as reasons underlying the gaps in the cascade [18]. However, the utility of any version of the cascade framework, either simple or extended, needs to be demonstrated using data to specifically populate HPCs for both individual and combination HIV prevention [27].

Recent studies have reinforced the importance of including individual-level motivation within the framework [25,28]. The inclusion of individual-level motivation distinguishes the Schaefer et al framework from other proposed frameworks such as that included in recent operational guidance from UNAIDS on creating HIV prevention cascades [29]. The UNAIDS approach adopts a more programmatic perspective with the cascade steps consisting of identifying a focus population and then measuring the reach/coverage, uptake/use and then correct/consistent use of primary HIV prevention methods [29].

### Collection and application of real-world data to populate the HIV Prevention Cascade

Several studies applying the Harare HPC to various priority populations have successfully populated the core steps of the HPC cascade [30], but these studies have reported lack of data availability when using the extended framework with explanatory sub-bars [30–32].

Despite an emerging consensus that the cascade should consist of initially defining a priority population, followed by measuring motivation, access and use within this population, population survey data collected specifically to populate and validate the cascade framework are lacking [27]. Before encouraging use of this HPC framework, it is necessary to understand and demonstrate the practicality of collecting data to specifically populate the cascade. The Manicaland General Population Open Cohort Study, an ongoing open cohort study in east Zimbabwe, has collected data to test the practical utility of the particular generic HPC proposed by LWG/Schaefer *et al* [18], to be known as the Harare HPC framework. The pilot survey tested a questionnaire module to capture data on HPCs across multiple primary HIV prevention methods: male condoms, female condoms, PrEP and VMMC.

### Validating the HIV Prevention Cascade Framework

The Harare HPC framework requires validation using data specifically collected to populate the framework. Collecting data via population surveys is crucial to understand the full picture of HIV prevention method use within the larger population compared to clinic-based programmes. Reviewing the pragmatic validity of the proposed HPC framework aims to assess whether the requirements of its specific intended use are fulfilled when populated with survey data. As emphasised repeatedly, a key feature of any cascade formulation is that it must be simple and practical to populate [17,18,25]. Therefore, the most parsimonious version of the HPC framework possible should be favoured when reviewing the validity of measures and survey tools to populate the framework whilst trying to maximise the information which can be gained from the framework. This will maximise the likelihood of the Harare HPC being adopted across national and sub-national programmes and research, as well as being the most feasible to include in population surveys such as Demographic Health Surveys (DHS).

Defining the priority population, the denominator of the HPC framework, is the first challenge to populating the cascade. Specifying a priority population for prevention is more complex than the definition of a starting population of the HIV treatment cascade [26]. The definition of the initial denominator of the population at risk will have a knock-on effect on the entire cascade [26]. Schaefer *et al.* define the priority population as the “population that could benefit from using a prevention method”, a definition which is open to adaptation according to the (national or sub-national) population of interest [18]. Within the context of Manicaland, work has been done to establish the definition of priority populations through a combination of literature review and analysis of sexual behaviours associated with HIV acquisition [18,33].

Following the definition of the priority population, the Harare HPC core framework consists of three main bars: motivation, access, and effective use of prevention methods [18]. Motivation captures an individual’s desire to use a prevention method. Access captures whether an individual is able to access a prevention method. Effective use describes the use of a prevention method required to prevent acquisition of HIV [18]. For the proposed Harare HPC framework to successfully describe the steps taken for an individual to effectively use HIV prevention, the steps leading to effective use must be predictive of effective use. Although the core cascade is designed to be generic and applicable across populations, validating the questionnaire module developed to populate the framework and the combinations of questions to measure each domain of the cascade is required to help promote use of the HPC framework.

Schaefer et al. stress the importance of individual-level motivation in prevention method use and therefore suggest that this should be the first step in the HPC [18]. Early exploratory analysis of the cascade has consistently indicated a very small drop between the motivation bar and the access bar. This raised questions about how the main bar for access was being defined and whether using a single question – “do you know a place you could access a prevention method” – was sufficient and identified a need to explore the overlap between motivation to use and access to prevention methods. This compounds a point raised during the Harare workshop that the HPC may not be as clearly linear as the treatment cascade, meaning the proposed order of motivation and access may be reversed or highly correlated [25]. This hypothesis requires testing using general population data. Schaefer *et al.* highlight that, given that both motivation and access are necessary for effective use, their order in in the cascade is unlikely to impact programmatic decisions. Regardless of access, an individual will not use a prevention method if they are not motivated to, and individuals may still experience barriers in their capacity to use prevention methods effectively [18]. However, if interventions to improve HIV prevention methods are designed based on gaps identified in the cascade then the HPC framework must effectively highlight these gaps – something which could be affected by the order of the bars [24]. Auerbach *et al.* also suggested that in priority populations which have very high coverage of access to a prevention method, ordering the cascade so that access comes first would give greater insight to motivation to use a prevention method when access is not an issue [26]. The different insights gained from the HPC framework when swapping the order of motivation and access however remain to be demonstrated.

The extended version of the Harare HPC framework includes sub-bars which act as explanatory variables hypothesised to explain the gaps in each bar of the cascade. The sub-bars apply to specific domains of the cascade and therefore it is hypothesised in the framework that these explanatory variables are associated with a lack of each domain. For example, explanatory variables hypothesised to explain a lack of motivation should be associated with a lack of motivation. Inclusion of these variables in the framework was based on extensive literature review and models of behaviour change [11], but it is useful to test these associations using data specifically collected to populate the HPC framework [18,34]. One key strength of this Harare HPC framework is how it allows the demonstration of the multiple multi-level barriers which individuals and populations experience.

Further to work that has been carried out so far, robust methods for defining and interpreting HPCs are required. It is necessary to validate:

1) The steps and sub-bars in the cascade framework
2) The questionnaire module developed to populate and measure the cascade framework

We aim to assess and refine the ability of the HIV Prevention Cascade and questionnaire module proposed by Schaefer et al. to identify potential targets for HIV prevention interventions using data from a general population survey through the following objectives:

1. Test the feasibility of populating the HIV Prevention Cascade draft formulation with questions in the prevention questionnaire tool collected in the Manicaland pilot survey
2. Test whether the motivation and access main bars of the cascade frameworks are predictors of effective use of HIV prevention methods
3. Contrast alternative ways of measuring each main bar in the cascade defined in the Manicaland HIV Prevention Cascade pilot survey questionnaire module
4. Compare the populations captured within each main bar of the HPC draft formulation when the order of the motivation and access bars are swapped
5. Test the validity of the sub-bars to explain why individuals are lost from the HPC and are not effectively using HIV prevention methods
6. Propose a final validated version of the HPC framework
7. Propose a minimum questionnaire module to populate the main bars and explanatory sub-bars of the HPC framework

## Methods & materials

### Data sources, study design and study setting

Data were taken from the Manicaland HIV Prevention Cascade Pilot Study collected in 2018–19, which was carried out in Manicaland Province in eastern Zimbabwe [35]. This pilot study was embedded within the Manicaland General Population Open Cohort Study; a long running cohort study in eastern Zimbabwe which spans a range of socioeconomic strata and urbanicities. Data from this cohort has regularly contributed to comparative and meta-analyses with other HIV cohorts across the region [36,37]. Data on sociodemographic characteristics, HIV knowledge, risk and prevention method use were collected in a pilot questionnaire designed specifically to populate the Harare HPC framework [38]. This represented the first dataset designed to populate and validate the Harare HPC framework.

Eligibility to participate in the individual questionnaire and HIV testing was established following the household questionnaire. Individuals were eligible if they were aged 15 years and above and had been resident in the household for the previous 4 nights. Out-migrants were not eligible for participation.

Among those eligible, all females aged 15–24 years and all males aged 15–29 years were invited to complete the individual questionnaire to ensure adequate representation of younger adults for an accompanying trial (carried out following the survey) [39]. Two thirds of females aged 25 years and over and males aged 30 years and over were randomly selected as eligible for participation in the individual questionnaire which is the standard sampling fraction for the Manicaland cohort [40]. Eligible individuals were invited to participate at the end of the household questionnaire. Ethical approvals for all survey activities were granted by the Medical Research Council of Zimbabwe (MRCZ/A/2243) and the Imperial College Research Ethics Committee (17IC4160). Written informed consent for collection and use of survey data, HIV testing and laboratory results was collected from all participants prior to their participation in the study. For participants aged under 18 years, assent was collected and informed parental consent was collected. Study recruitment and participation took place between 9^th^ July 2018 and 14^th^ December 2020. Data was collected using Open Data Kit (ODK) which helped to maintain data quality. The questionnaire within ODK incorporated pre-programmed skip patterns, range and consistency checks, enforced data types, and compulsory responses to minimize entry errors and missing data. For sensitive questions, the Informal Confidential Voting Interview (ICVI) methods was employed, allowing participants to record responses privately without disclosing them to the interviewer [41]. Questionnaires were delivered in Shona: the local language of the study sites.

### Manicaland HIV Prevention Cascade Questionnaire module

The Manicaland HPC pilot study implemented a draft questionnaire module containing questions to populate HIV prevention cascades for male condoms, female condoms, VMMC and PrEP [38]. Questions were proposed following a stakeholder consultation during the Harare Workshop [25] and developed into HIV prevention modules of an individual questionnaire [25]. As part of this pilot survey, PrEP adherence laboratory testing was conducted on a sub-sample of young females reporting current or recent PrEP use [33,42]. Current PrEP adherence was defined by a concentration of Tenofovir above 0.7pM per dried blood spot (DBS) punch [43]. All other data collected as part of the questionnaire module were self-reported, including that on sexual risk behaviour and all prevention method use. As mentioned early, ICVI methods were used to reduce social desirability bias.

### Definition of the priority population

To maximise the sample size available for validation purposes, a broad definition of a priority population was used: HIV-negative participants aged 15–54 years who reported one or more sexual risk behaviours for HIV acquisition in the last 12 months. HIV status was determined by either provider-initiated testing and counselling (PITC) or laboratory-based testing of DBS specimens [33]. Sexual risk behaviours were selected based on literature review and an HIV incidence analysis of the Manicaland general population cohort [33]. Risk behaviours included were having multiple partners in the last 12 months; concurrent partners at the time of interview; recent transactional sex in the last month with any of the last three partners; and having at least one non-regular partner in the last 12 months.

### Populating the HIV Prevention Cascade

Definitions of each bar and sub-bar of the HIV prevention cascade for each prevention method are listed in S1-S3 Tables.

Outcomes were coded as binary variables and the bar height was calculated as this proportion within the target population:

- 0 = does not meet criteria for that bar/sub-bar
- 1 = does meet criteria for that bar/sub-bar

For any main bar (being motivated to use, having access to or effectively using a prevention method) this was coded as:

- 0 = individual is not motivated/does not have access to/is not using a prevention method
- 1 = individual is motivated/does have access to/is using a prevention method

For barrier bars (for example as knowledge/lack of knowledge as a barrier to prevention method use) this was coded as:

- 0 = individual does not experience a lack of knowledge as a barrier to prevention method use
- 1 = individual does experience a lack of knowledge as a barrier to prevention method use

An individual only fell within a barrier bar if they did not meet the criteria to fall within the main bar, so the cascade takes the perspective of those who have the positive motivation/access/effective use, and then seeks to understand the barriers for those who do not fall within a main bar. It was assumed that all those who effectively use a prevention method were also motivated to use and had access to each HIV prevention method. The HPC framework was populated as a conditional cascade; each step was conditional on the previous step and therefore an individual must be in the previous step of the cascade in order to be allowed to experience the following step. For example, an individual must be motivated to be considered in the denominator for the access bar.

Each main bar of the cascade was calculated as a proportion:


$$Proportion\;motivated=\;\frac{\begin{array}{c} number\;of\;individuals\;reporting\;to\;be\;motivated\;\\ to\;use\;a\;prevention\;method \end{array}}{number\;of\;individuals\;in\;priority\;population}$$

$$Proportion\;with\;access=\;\frac{\begin{array}{c} number\;of\;individuals\;reporting\;to\;be\;motivated\;to\;use\;\\ and\;have\;access\;to\;a\;prevention\;method \end{array}}{number\;of\;individuals\;in\;priority\;population}$$

$$Proportion\;effectively\;using=\;\frac{\begin{array}{c} number\;of\;individuals\;reporting\;to\;be\;motivated\;to\;use,\\ have\;access\;to\;and\;be\;effectively\;\\ using\;a\;prevention\;method \end{array}}{number\;of\;individuals\;in\;priority\;population}$$

95% confidence intervals for each main bar proportion were calculated for the main bars of the HPC framework.

Explanatory sub-bars were populated for individuals who met the conditions to continue through to that domain of the cascade, but did not report the main bar for this domain positively. For example, to be in the access bar individuals must report to be motivated. Explanatory barriers to motivation were populated for those who were in the priority population but did not report to be motivated. Barriers to access were populated for those who did report motivation but did not report access. Barriers to effective use were populated for those who reported motivation and access but did not report to be using a prevention method.

### Combination prevention method use

The combination HPC framework was populated by classifying whether individuals in the priority population used at least one of the prevention methods:

a. Males – VMMC and/or male condoms
b. Females – PrEP and/or male condoms

Female condom use was not included when exploring combination prevention method use, due to very low reported numbers. When calculating the main bars (e.g., motivation), individuals had to report affirmatively for those bars for at least one prevention method. This calculation built on definitions of individual prevention method use and so the combination cascade remained conditional specific to individual prevention method use, e.g., an individual could not be part of the combination motivated bar based on male condom motivation and then part of the combination access bar based on VMMC access.

**Fig 1:**
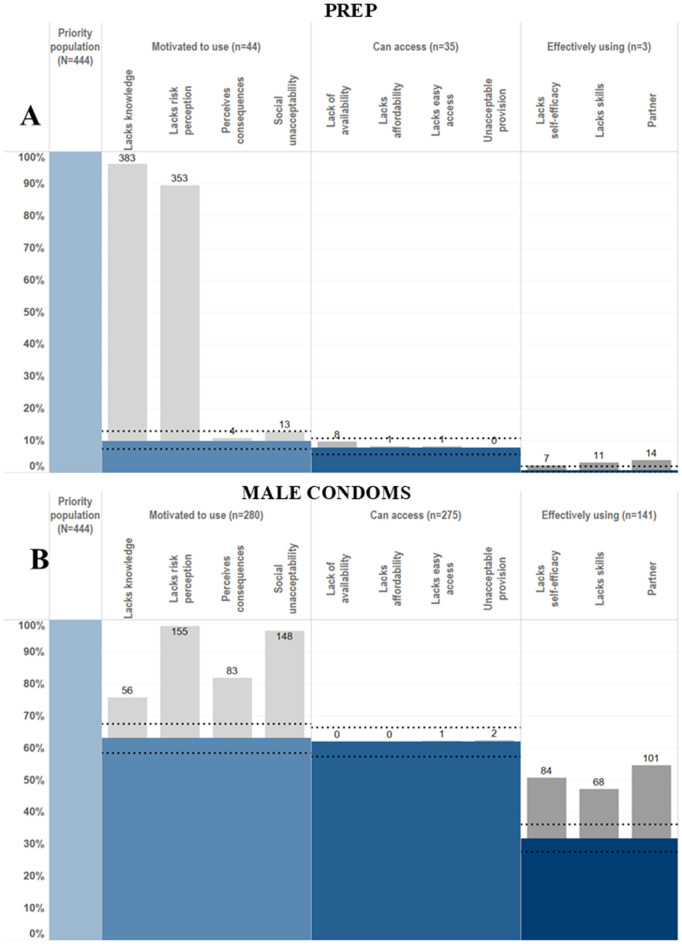
HIV prevention cascades for PrEP and male condom use in females in the priority population.

Individuals were included within combination explanatory sub-bars when they did not fall into a main bar (e.g., motivation) for any prevention method. The sub-bars were assigned to the furthest possible point along the cascade, with the end point of any prevention method use in mind. For example, if an individual was motivated to use male condoms but lacks motivation to use VMMC, they continued to the access domain of the framework because they were motivated to use at least one method. At this point, if they reported a lack of access to male condoms, then explanatory barriers to this access should be reported within the cascade. Please see Fig 1 as an example of how the Harare HPC framework is visualised.

### Measuring validity of the HPC framework

Hypotheses tested within each objective are listed in Table 1. Accepting or rejecting each hypothesis is not solely based on statistical outcomes but also on the practicality and feasibility of each part of the HPC framework, and considering the previous justifications (which are grounded in the literature and social cognitive theories) for each part of the HPC framework as set out by Schaefer et al, the LWG and during the Harare Workshop.

**Table 1:** Hypotheses tested within each objective of the validation process and the output evaluated within each objective.

Objective		Hypothesis	Output for evaluation
1	Test feasibility of populating the HIV Prevention Cascade draft formulation with questions in the prevention questionnaire tool collected as part of the Manicaland pilot survey	It is possible to populate the HPC framework with questions developed as part of the questionnaire module of the Manicaland HPC survey	Individual and combination cascades including measurements of the main and sub bars
2	Test that each of the main bars within the steps of the cascade frameworks are predictors of effective use of HIV prevention methods	Motivation is associated with effective use of HIV prevention methods	Logistic regression measuring association between an exposure of being motivated or not and separate outcomes of male condom use, PrEP use and VMMC
		Access is associated with effective use of HIV prevention methods	Logistic regression measuring association between an exposure of having access or not and separate outcomes of male condom use, PrEP use and VMMC
3	Contrast ways of measuring each of the main bars of the cascade defined in the Manicaland HIV Prevention Cascade pilot survey questionnaire module	There is no difference in the populations captured within each bar of the cascade when using the simplest measures included as part of the Manicaland HPC pilot survey questionnaire module	HPCs of simple measure vs Likert scale cut off Test of difference in proportions of population in each main bar of HPC with simple vs alternate definitions Compare self-reported use of prevention with alternative measures: condom use at last sex vs all sex and intention at next sex, PrEP adherence measures, clinic confirmed VMMC
4	Compare populations captured within each main bar of the HPC draft formulation when the order of the motivation and access bars are swapped	There is no difference in the drop offs of the cascade when the access bar is swapped with the motivation bar	HPC figures with alternate order for each prevention method Test of difference in proportions of population lost in each step of the HPC with each order (motivation>access>use/access>motivation>use)
5	Test the validity of the sub-bars to explain why individuals are lost from the HPC and are not effectively using HIV prevention methods	The explanatory factors are associated with a lack of effective use of HIV prevention methods	Regression of explanatory factors with each of male condom use, PreP use and VMMC
		The explanatory factors are associated with the corresponding main bar	Regression of explanatory factors with each main bar, e.g., motivation sub bars with motivation
		The sub-bars additively explain the gaps in the cascade and are sufficient to explain reasons for lack of use of different HIV prevention methods	% of each gap explained by sub-bars & comparison with explanatory factors for lack of prevention method use identified from qualitative aspects of the study
6	Propose a final validated version of the HPC framework		Final formulation of HPC framework
7	Propose a minimum questionnaire module to populate the main bars and explanatory sub-bars of the HPC framework & compare the questionnaire module to the questions available in routinely collected questionnaire modules		Minimum questionnaire module with comparison to similar questions available from routinely collected data

### Objective 1

The feasibility of populating the HPC framework was tested using data collected from the Manicaland pilot questionnaire modules. Full cascades were populated for male condoms, PrEP and combination prevention in the female priority population and male condoms, VMMC and combination prevention in the male priority population.

### Objective 2

Logistic regression was used to test the association of motivation and access as predictors of the outcome of effective use of HIV prevention methods. Regressions were fitted separately for each prevention method (male condoms, PrEP and VMMC), adjusted for 5-year age group and site type, and stratified by sex. Female condom use was not explored here due to very low use reported. P-values were interpreted as indicative of the strength of evidence rather than as strict thresholds for statistical significance and no multiple testing corrections were applied.

### Objective 3

Multiple potential questions to populate each main bar of the HPC framework were included in the Manicaland pilot questionnaire module. The simplest combination of questions used to populate the cascade was considered the most favourable measure to ensure the HPC framework is as feasible as possible to routinely collect data on. Each of the main cascade bars was calculated using the simplest (primary) measure and then also using more complex (alternate) measures, as detailed in S1-S3 Tables, for each prevention method and stratified by sex. Throughout this analysis, the definition of the main cascade bars included those effectively using each prevention method. Differences in the proportions captured within each bar of the cascade using simple versus alternate measures were compared using a two-sample proportion test. P-values for two-tailed tests of significance between the proportions were used to assess the hypothesis that there is a difference in proportions identified through the simple versus alternative measure. P-values were interpreted as indicative of the strength of evidence rather than as strict thresholds for statistical significance and no multiple testing corrections were applied. Self-reported use of prevention methods was compared with alternative measures—self reported condom use at last sex versus condom use throughout all sex in the last year, self-reported PrEP use versus laboratory confirmed PrEP use and self-reported VMMC versus clinic confirmed VMMC [33]. Logistic regression was used to test the association between these measures of prevention method use. The proportion of individuals with overlap between reporting a positive bar and also reporting barriers to each domain of the cascade were calculated and presented using Venn diagrams, e.g., those who report to be motivated to use a prevention method but also report barriers to motivation.

### Objective 4

Cascades were populated for each prevention method, stratified by sex, using the alternate order of the main bars (access > motivation > effective use). The overall proportions lost from the cascade between the starting point of the priority population to the effective use bar with each order of the bars were compared. The proportions lost at each step of the cascade were compared for each order of the main bars using z-tests. If interventions to improve HIV prevention methods are designed based on gaps identified in the cascade then the HPC framework must effectively highlight these gaps, and therefore the version of the cascade which would best demonstrate these gaps

### Objective 5

The association of explanatory factors with use of each prevention method was tested using multivariate logistic regression, adjusted for 5-year age group and site type, and stratified by sex. We follow prior cascade models in using the term ‘lack’ to reflect participants’ self-reported perceived barriers at the motivation, access, or use steps of the cascade (e.g., lack of knowledge). The association of each explanatory factor with each domain of the cascade was tested using multivariate logistic regression, adjusted for 5-year age group and site type. This was carried out separately for each prevention method and stratified by sex, to test if the explanatory factors correctly explained the gap in the cascade they had been assigned to in the proposed HPC framework. It was anticipated that multiple explanatory factors would be experienced by individuals and that they would be collinear, which is in fact a key strength of the HPC framework. Therefore, these explanatory factors were be considered individually, and collinearity was not be explored. The total proportion of each gap explained by the explanatory sub-bars was measured. Where the gaps were not fully explained additively by the explanatory sub-bars, the results were compared with explanatory factors for lack of prevention method use identified from individual interviews and focus groups from qualitative parts of the study. Factors identified from qualitative work but not covered by the HPC framework were reviewed [33]. P-values were interpreted as indicative of the strength of evidence rather than as strict thresholds for statistical significance and no multiple testing corrections were applied

### Objectives 6 & 7

Based on results from these analyses, a final version of the HPC framework was proposed. A questionnaire module was recommended based on the minimum questions required to populate a simple (main bars only) and extended (including explanatory sub-bars) HPC framework. The simplest measures were favoured where possible to maximise the feasibility of use of the HPC framework across multiple settings and data sources including population level surveys.

All statistical analyses were carried out using Stata/MP 17.0. Data visualisation was carried out using Tableau 2022.4.

## Results

### Participant characteristics

A total of 9803 individuals aged 15 years and above completed the individual questionnaire (females n = 5729; males n = 4074), representing 77% of those identified as eligible for participation from the household census. Of all individuals completing the individual questionnaire, 9339 (95%) had an HIV result, either from PITC (n = 7715) or DBS laboratory testing (n = 1624). Among individuals with an HIV test result, 90% (8,404) were HIV negative. Of the 8,404 HIV-negative individuals, 6,307 (75%) self-reported having initiated sexual activity, and 5,223 (83%) of these were aged 15–54 years. Among those sexually active 15–54-year-olds, 14% of females and 28% males reported ≥1 HIV sexual risk behaviour resulting in priority populations of 575 and 444 HIV negative males and females respectively.

Participants’ demographic characteristics are summarised in S4 Table. Participants were distributed across urban, peri-urban, estate, and rural settings, with rural residence more common among women and estate residence more common among men (S4 Table). Educational attainment was generally high, with the majority reporting secondary or higher education, particularly among men (approximately 89–91%). Marital status profiles differed by sex: most women were currently married, whereas men were more frequently never married. Within the priority populations, divorce or separation was reported more often among women compared to men. Socioeconomically, the largest proportion of participants fell within the second poorest quintile, while very few were classified in the least poor group.

*Objective 1 - Test feasibility of populating the HIV Prevention Cascade draft formulation with questions in the prevention questionnaire tool collected in the Manicaland pilot survey*

**Fig 2:**
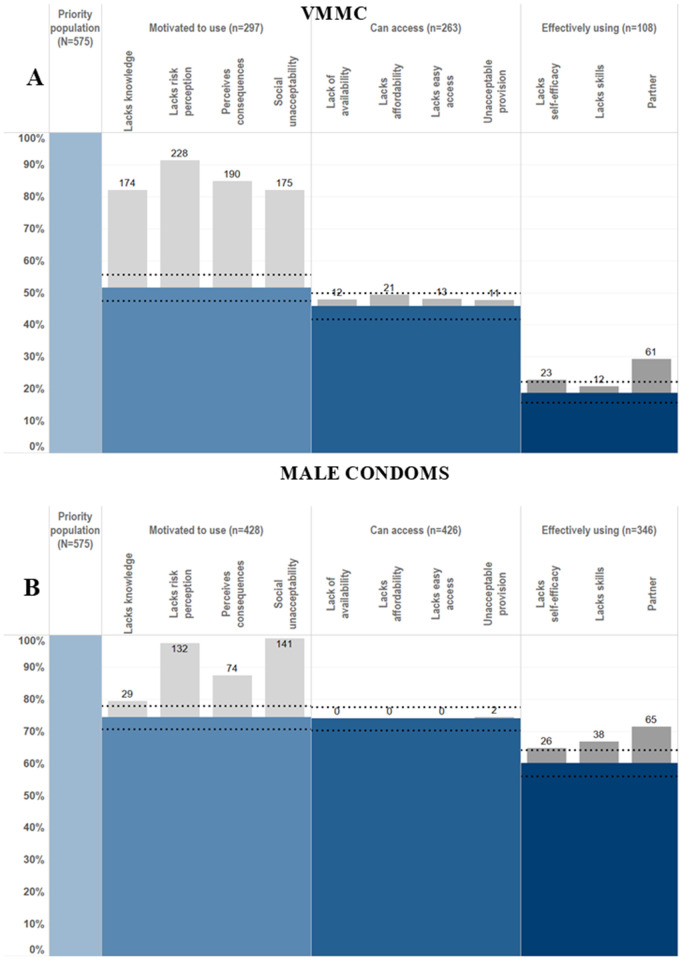
HIV prevention cascades for PrEP and male condom use in females in the priority population.

It was possible to create HPCs for PrEP and male condom use among females (Fig 1) and VMMC and male condom use among males (Fig 2) in the priority population using data collected within the Manicaland pilot questionnaire module. Levels of motivation, access and use of each prevention method could be assessed for each priority population and compared across prevention methods. Of women in the priority population, 63% were motivated to use, 62% were motivated and had access to and 32% reported motivation, access, and male condom use. PrEP use was lower: 10% were motivated to use, 8% had access to and <1% reported PrEP use. Fifty-two percent of men in the priority population were motivated to have VMMC, 46% reported access and 19% reported full medical male circumcision. Male condom use rates by men was higher than VMMC: 74% were motivated to use, 74% had access to and 60% reported male condom use.

**Fig 3:**
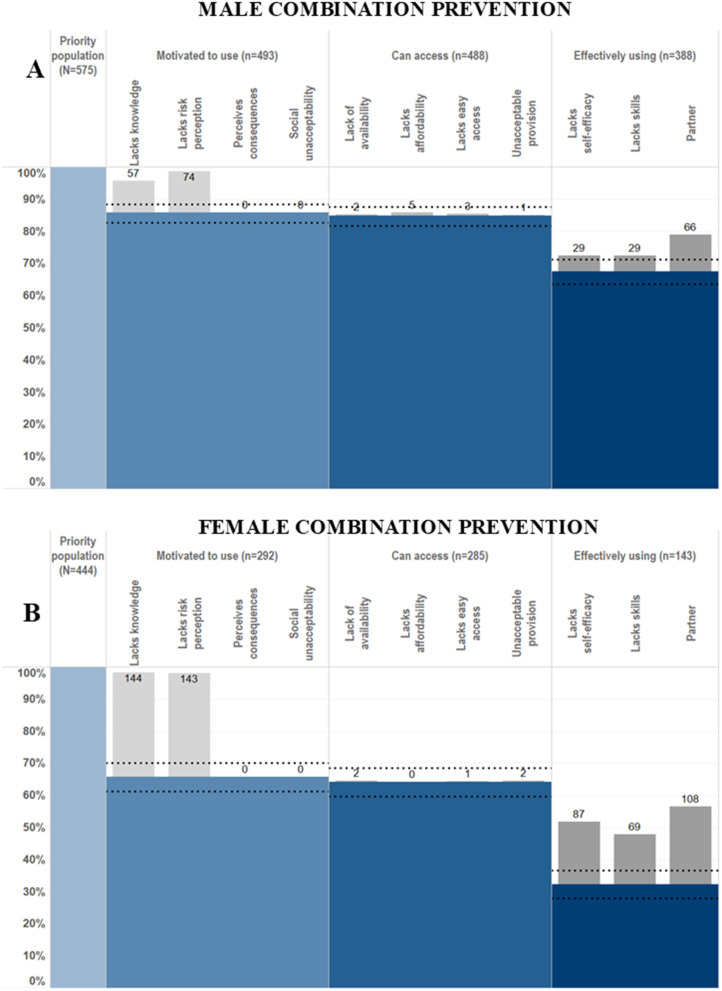
HIV prevention cascades for HIV combination prevention: use of VMMC or male condoms in males and PrEP or male condoms in females.

Where gaps in the cascade were identified, it was possible to populate sub-bars explaining the gaps in the cascade and barriers to progressing through the cascade. Barriers observed include lack of knowledge, lack of risk perception, perceived negative consequences of use and partner resistance. It was also possible to populate combination prevention cascades (Fig 3). Sixty-seven percent of men and 32% of women reported using at least one prevention method. It was possible to populate the sub-bars to understand explanatory barriers to use. However, it was not possible to see which prevention method the barriers were relating to, which prevention methods were preferred, or the proportion of people using multiple prevention methods. Some barriers, such as a lack of risk perception and a lack of knowledge of all prevention methods, could still provide some insight into barriers to combination prevention method use.

*Objective 2 - Test that each of the main bars of the cascade framework are predictors of effective use of HIV prevention methods*

Logistic regression models, adjusted for site type and 5-year age-group, were used to measure the association between motivation and prevention method use, then access and prevention method use (Table 2). Among both women and men, motivation to use condoms (women: odds ratio (OR)=26.83, 95% CI 11.73-61.35, men: OR=8.36, 95% CI 5.58-12.53) and access to male condoms (women: OR=17.90, 95% CI 8.91-35.97), men: OR=8.33, 95% CI 5.57-12.44) were strongly associated with use of male condoms, indicating that these are necessary components of the steps of the HIV prevention cascade. For PrEP, it was not possible to calculate odds ratios for associations of motivation and access with PrEP use due to small numbers of people in the priority population reporting PrEP use, all of whom reported motivation and access. Motivation for (OR=0.06, 95% CI 0.02-0.15) and access to VMMC were significantly associated with lower odds of having had VMMC. This is likely due to the one-off nature of the procedure and the phrasing of the questions: 95% of people reporting VMMC responded that they were not motivated to get VMMC or did not know somewhere to access VMMC if they did want to access it, probably because they had already had the procedure. If the HIV prevention cascade was populated without applying this ‘effective use assumption’—that all reporting prevention method use must be motivated and have access to that prevention method—then inaccurate levels of motivation and access would be captured in the cascade.

**Table 2:** Associations between motivation and access with prevention method use, adjusted for site type and 5-year age group.

	Motivation		Access	
n (%)	OR (95% CI)	n (%)	OR (95% CI)
PrEP (Female)	44 (9.9)	*collinear*	4 (0.9)	*collinear*
VMMC (Male)	252 (43.8)	0.06 (0.02-0.15)	228 (39.7)	0.09 (0.03-0.22)
Male condoms (Female)	280 (63.0)	26.83 (11.73-61.35)	183 (41.2)	17.90 (8.91-35.97)
Male condoms (Male)	428 (74.4)	8.36 (5.58-12.53)	391 (68.0)	8.33 (5.57-12.44)

*Objective 3 - Contrast alternative ways of measuring each of the main bars of the cascade defined in the Manicaland HIV Prevention Cascade pilot survey questionnaire module*

**Fig 4:**
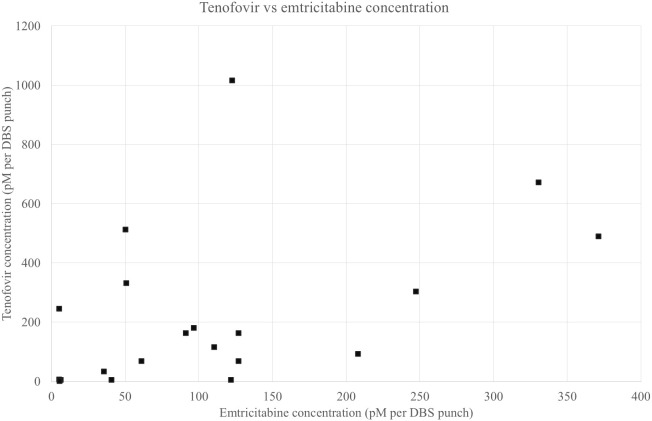
Tenofovir and emtricitabine concentrations from DBS testing in females aged 15-24 years reporting current or recent PrEP use.

Simple versus alternative (more complex) ways of measuring the main steps of the HPC (S1-S3 Tables) were calculated, and the proportions of the population captured using each definition were compared using two-tailed two sample test of proportions (Table 3). There were no significant differences between the populations captured in the simple versus alternative measures for motivation or access across all prevention methods explored. It was not possible to compare VMMC use to an alternate measure due a lack of data on other ways of confirming VMMC. Contrasting ways of defining male condom use were evaluated. The simple measure - self-reported current male condom use - was compared firstly with self-reporting using male condoms throughout last sexual encounter, and, secondly, self-reporting using condoms at every sexual encounter for as long as an individual has been sexually active. Significantly fewer (p < 0.001) men and women reported using the simple measure compared to using condoms at every sexual encounter in both men and women, with a decrease from 32% to 6% in females and 60% to 16% in males. When comparing reporting to use a condom currently with self-reporting condom use at last sex, significantly fewer men reported condom use at last sex (p < 0.001) with a decrease from 60% to 43%. There was no significant difference in the female populations captured (p = 0.432). Self-reported current PrEP use was compared to another self-reported measure of using PrEP all or most days in the last month and no difference was observed in the populations of women captured (p = 0.999). Testing for the presence of tenofovir and emtricitabine was carried out on DBS collected in young women reporting current or ever PrEP use across the study (N = 18) (Fig 4). All DBS samples had concentrations of Tenofovir above the threshold for PrEP adherence (0.7pM per DBS).

**Table 3:** Comparison proportions of motivation, access, and use of prevention methods with simple vs alternative measures and p-values for differences in proportions using 2-sample test of proportions.

	Simple measure	Alternative measure	
**% (95% CI)**	**% (95% CI)**	**p-value**
**Motivation**			
Male condoms - female	61.5 (56.9-65.9)	58.8 (54.1-63.3)	0.411
Male condoms - male	63.5 (59.5-67.3)	62.3 (58.2-66.1)	0.669
VMMC - male	51.7 (47.6-55.7)	51.0 (46.9-55.0)	0.813
PrEP - female	9.9 (6.9-12.3)	9.9 (7.5-13.1)	0.999
**Access**			
Male condoms - female	59.5 (54.8-63.9)	57.2 (52.5-61.7)	0.634
Male condoms - male	62.8 (58.7-66.6)	62.3 (58.2-66.1)	0.999
VMMC - male	27.8 (24.3-31.6)	24.3 (21.0-28.0)	0.179
PrEP - female	7.6 (5.1-10.0)	6.8 (4.8-9.5)	0.999
**Effective use**			
Male condoms - female	31.8 (27.6-36.2)	5.6 (3.8-8.2)	<0.001
Male condoms at last sex* - female	31.8 (27.6-36.2)	34.2 (30.0-38.8)	0.432
Male condoms - male	60.2 (56.1-64.1)	15.8 (13.1-19.0)	<0.001
Male condoms at last sex* - male	60.2 (56.1-64.1)	43.0 (39.0-47.0)	<0.001
VMMC - male	18.8 (15.8-22.2)	n/a	n/a
PrEP - female	0.7 (0.2-2.1)	0.7 (0.2-2.1)	0.999

**Fig 5:**
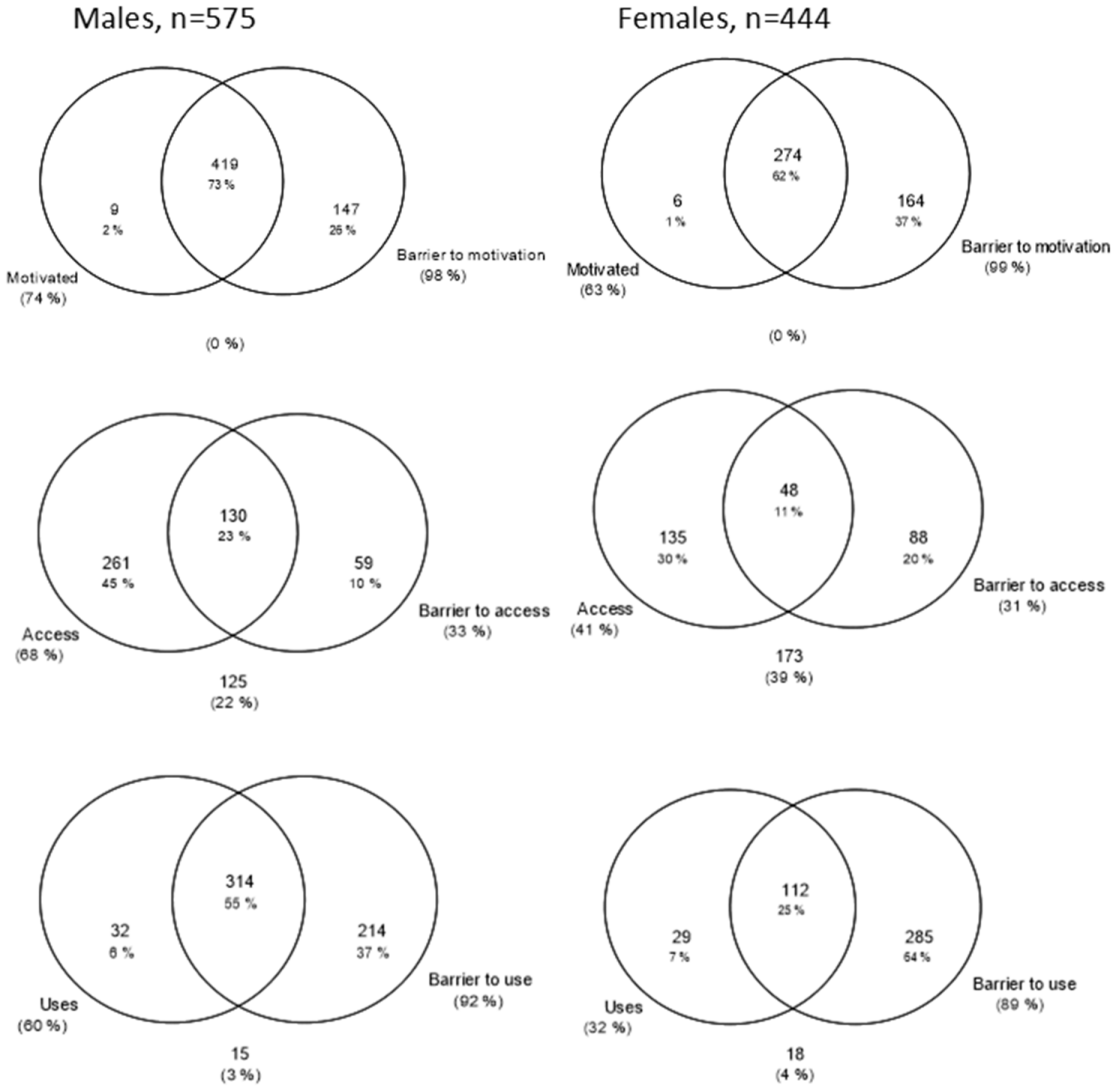
Venn diagrams of overlaps between each bar of the cascade and reporting at least one barrier to that bar for male condom use in males and females.

**Fig 6:**
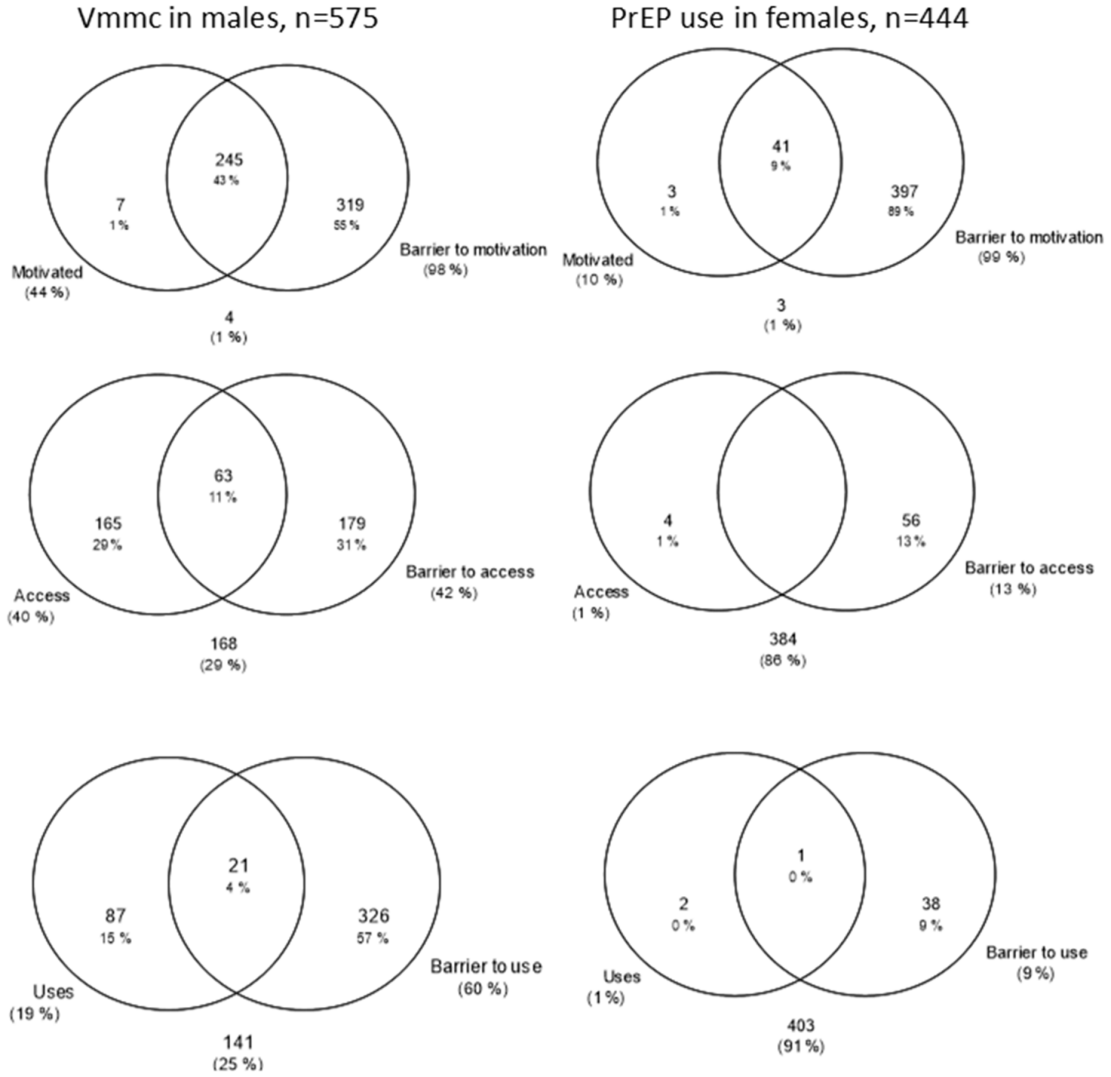
Venn diagrams of overlaps between each bar of the cascade and reporting at least one barrier to that bar for VMMC in males and PrEP use in females.

Overlaps between populations reporting main bars (e.g., motivation) and reporting barriers to those bars were assessed for the whole priority population without the conditional nature of the cascade (Figs 5–6). Seventy-three percent of men and 62% of women reported motivation to use male condoms but experiencing a barrier to use (Fig 5). Ninety-eight percent of people reporting use of male condoms also report a barrier to motivation. Twenty-three percent of the male priority population and 11% of the female priority population report both access to male condoms and also barriers to access. Fifty-five percent of males and 25% of females in the priority population report both use of male condoms and at least one barrier to male condom use. Conversely, for PrEP, women reported only small overlaps between motivation, access (Fig 6). Men reported larger overlaps in motivation to have VMMC and reporting a motivation related barrier (43%) (Fig 6). Eleven percent reported both access and an access related barrier and only 4% reported to have VMMC and also experience a barrier to VMMC.

*Objective 4 - Compare populations captured within each main bar of the HPC draft formulation when the order of the motivation and access bars are swapped*

**Fig 7:**
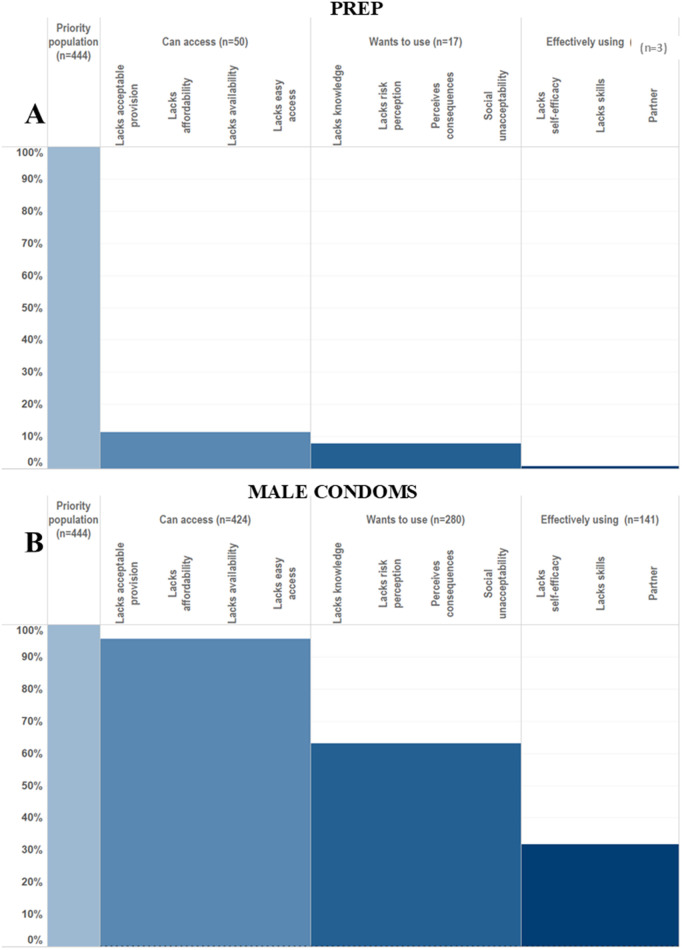
HIV prevention cascade with the order of Access, Motivation and Use, for PrEP and male condom use in females.

**Fig 8:**
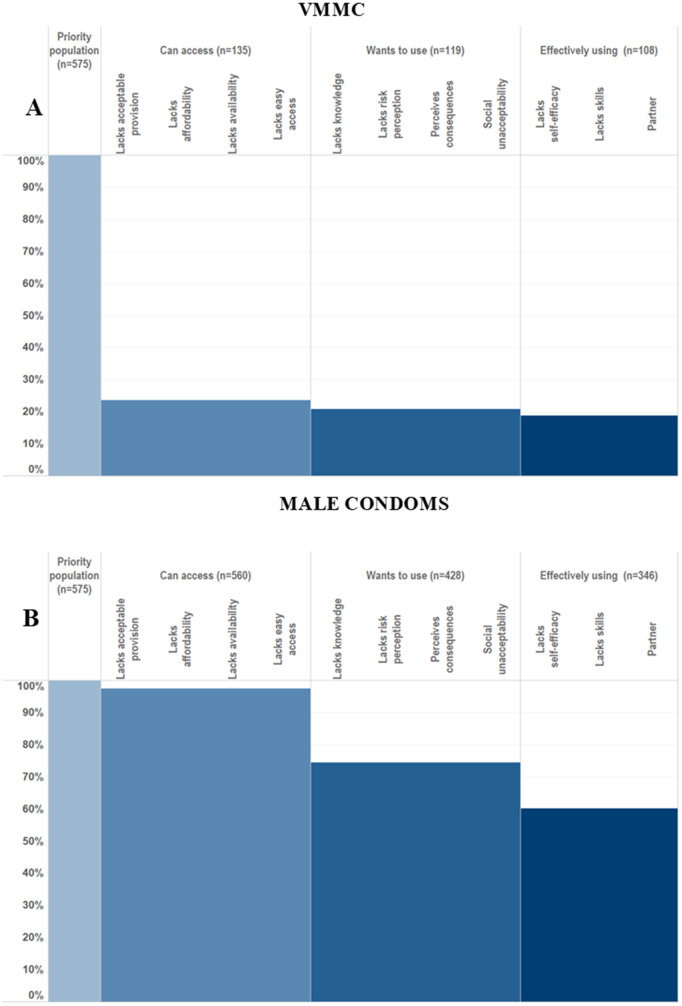
HIV prevention cascade with the order of Access, Motivation and Use, for VMMC and male condom use in males.

HIV prevention cascades were populated, for the main bars only, with the order of the main bars swapped and access being the first step of the cascade (Figs 7–8). The proportions of the population reaching the ‘use’ bar of the cascade were unchanged across all prevention methods. As with the first version of the cascade (Fig 1B), only a very small proportion of the priority population reported a lack of access to male condoms (5%) (Fig 7B). There was still a large gap in motivation with a large drop between the access and motivation bars: 34% of those with access were not motivated to use male condoms. Access to PrEP was low, and lack of access was the largest gap in the PrEP cascade in women, with 89% of the priority population lost from the cascade here. The drop was smaller between the access and motivation bars of the cascade with 66% of those with access reporting a lack of motivation to use PrEP (7% of the entire priority population).

Seventy-seven percent of the male priority population reported a lack of access to VMMC (Fig 8A). The drop was small, 3%, between access to motivation. The drop was also small between the motivation and use bars: 2% of the total priority population. This is considerably smaller than the equivalent drops using the alternative order (Fig 3A) where 27% of the total priority population were lost from the second to third step. Only 2.6% of the male priority population reported a lack of access to male condoms (Fig 8B): a very small gap in access which is similarly observed using the alternative order (Fig 2B). Twenty-four percent of men in the priority population with access to male condoms were not motivated to use them, representing 23% of the total priority population. Nineteen percent of men with access and motivation were not using male condoms. Fourteen percent14% of the overall priority population were lost from the motivation to use step here; the same proportion as lost from the access to use step in the original order of the HIV Prevention Cascade.

*Objective 5 - Test the validity of the sub-bars to explain why individuals are lost from the HPC and are not effectively using HIV prevention methods*

**Fig 9:**
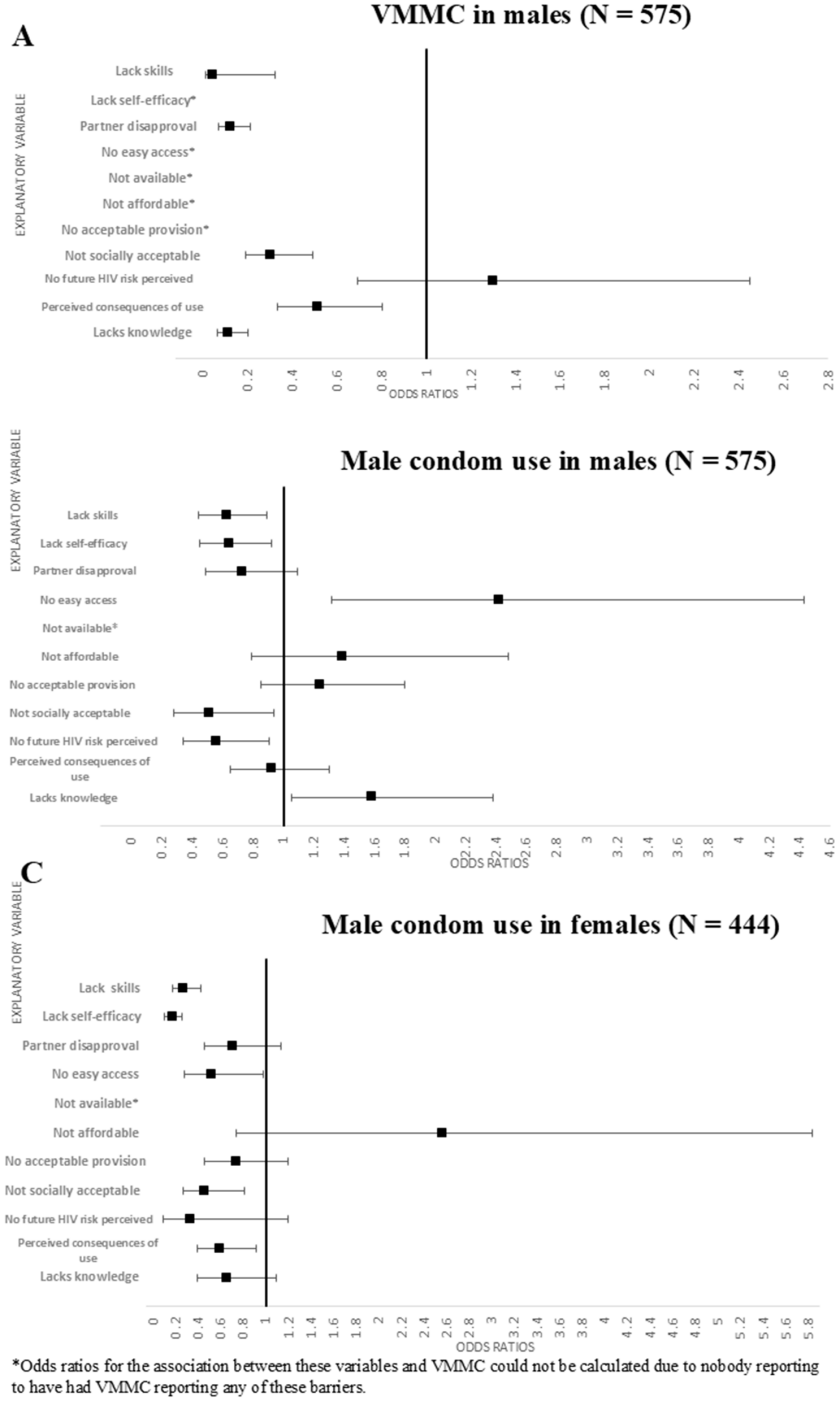
Associations of explanatory barriers with male condom use in females and male condom use and VMMC in males, adjusted for 5-year age group and site type.

Logistic regression models, adjusted for 5-year age group and site type, were used to assess the association between each of the hypothesised barriers in the HPC framework and use of each prevention method (Fig 9). Associations of PrEP related barriers with PrEP use were not calculated due to the very small numbers (n = 3) reporting PrEP use.

**Fig 10:**
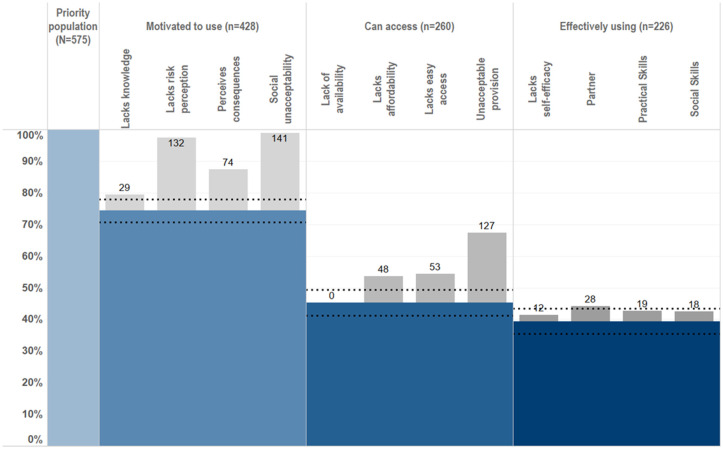
Final proposed formulation of the HIV Prevention Cascade, populated with data on male condom use in men.

Odds ratios for the association of VMMC with lack of self-efficacy, easy access, availability, affordability, and acceptable provision could not be calculated because nobody reporting VMMC reported any of these barriers. Almost all other hypothesised barriers were significantly associated with lower odds of having VMMC (Fig 10A). Lack of skills to negotiate with partner about having VMMC and partner disapproval were significantly associated with reduced odds of having VMMC: OR = 0.04 (95% CI: 0.01-0.32) and OR = 0.12 (95%CI: 0.07-0.21), respectively. Lack of social acceptability (OR = 0.3 (95% CI:0.19-0.49), lack of knowledge about HIV prevention benefits of VMMC (OR = 0.11, 95% CI: 0.06-0.20) and perceived negative consequences (OR = 0.51, 95% CI: 0.33-0.80) were all associated with reduced odds of VMMC among men in the priority population.

The only access related barriers significantly associated with lower odds of male condom use among men (Fig 9B) or women (Fig 9C) was lack of easy access in females (OR = 0.52 95% CI: 0.28-0.98). Lack of practical and socials skills and self-efficacy in using male condoms were associated with lower odds of male condom use in men and women. The motivation related barriers associated with lack of male condom use were different between men and women. In men, a lack of social acceptability (OR = 0.52 95% CI: 0.28-0.94) and a lack of future risk perception (OR = 0.56, 95% CI: 0.34-0.91) were the motivation related barriers associated with lack of male condom use. In women, a lack of social acceptability (OR = 0.46, 95% CI: 0.27-0.81) and perceived negative consequences of use (OR = 0.59, 95% CI: 0.39-0.91) were the motivation related barriers associated with lack of male condom use.

## Discussion

### Summary

This validation exercise demonstrated that the draft HIV Prevention Cascade framework could be feasibly populated for multiple prevention methods—male condoms, PrEP, VMMC, and combination prevention—using data from the Manicaland pilot survey. Across methods, motivation and access generally operated as intended: both were strongly associated with male condom use for men and women; PrEP motivation and access were low, and use was rare; VMMC showed high reported motivation but limited access, with the one-off nature of the procedure affecting how motivation and access were reported; and combination prevention showed substantial uptake among men (67%) but more limited use among women (32%). Sub-bars highlighted key barriers across methods, including lack of knowledge, limited risk perception, perceived negative consequences, and partner-related barriers. High overlap was observed between individuals reporting motivation/access and simultaneously reporting barriers, indicating that the framework is sensitive to nuanced self-reported challenges. Alternative definitions of motivation, access, and use generally did not change the populations captured, and swapping the order of motivation and access added little interpretive value. Together, these findings support the feasibility and face validity of the cascade framework, while illustrating important differences in motivation, access, and effective use across prevention methods.

### Strengths

Using the questionnaire modules piloted in this study, it was possible to define the priority population based on questions on sexual risk behaviours for HIV acquisition and then assess the HIV prevention cascades for this priority population in men and women for both individual and combination prevention method use. This was done using general population survey data which is the intended data source for this Harare HPC framework. Main bars of the cascades for both individual and combination prevention could be populated, giving insight into levels and gaps of motivation, access, and use of male condoms, VMMC and PrEP, as well as overall levels of VMMC and male condom cascades for men and PrEP and male condom cascades for women.

This demonstration of the feasibility of applying an HIV prevention cascade framework to combination primary HIV prevention is one of the first instances of such analysis [32]. In this analysis, the outcome of combination prevention considered was use of at least one prevention method. Depending on the priority population and the research question, combination cascades could be constructed with an outcome of motivation to use, access to and use of 2 or more prevention methods together where multiple prevention methods are recommended in tandem to provide better protection, e.g., VMMC and male condoms. Additionally, overall combination prevention cascades could be constructed in which all different combinations of prevention methods are considered. This version of the cascade framework allows this flexibility in application, provided the data are available. Application of the framework to combination prevention was one of the reasons highlighted by Schaefer *et al*. in the need for a standardised framework [18].

Motivation to use and access to male condoms were strongly associated with increased odds in condom use in both men and women, supporting the theory behind the inclusion of these key steps in the HPC framework. Due to the small numbers reporting PrEP use, motivation to use and access to PrEP were collinear with PrEP use. There were no significant differences in the populations captured by the simple versus alternative measures of motivation and access for VMMC, male condoms or PrEP, indicating that the simplest methods of defining motivation and access are sufficient to populate the HPC framework main bars.

Although Schaefer *et al*., and conclusions of the Harare workshop, argued that motivation and access are likely to be highly correlated [25] and the order of the cascade framework will not make much difference to programmatic decision making [18], swapping the order of the framework could mean that there are smaller drops offs observed in the cascade and therefore the areas identified as having the most potential to be improved by interventions could vary depending on the order of motivation and access. In this analysis, swapping the order of the motivation and access bars did not improve the information gained (i.e., larger gaps from which to identify barriers as targets for interventions) from the gaps between bars, and in the case of VMMC, actually meant less information about gaps in motivation could be gained from the cascade framework as most people were lost from the cascade in the first (access) bar.

Most barriers in the HPC were associated with a lack of effective use, supporting the theory behind the development of the framework in which the barriers were selected following preliminary analysis, literature review and behavioural theory [18,11]. These barriers identified through quantitative analyses are also supported by qualitative findings carried out within the same population [44,45]. Barriers to VMMC identified through qualitative focus groups and individual interviews included pain, fear of side effects and health consequences which pertain to the perceived consequences barrier of the cascade [45]. Barriers to PrEP use identified through these activities included a lack of knowledge and availability; barriers which were also indicated from the cascade analysis [44].

### Weaknesses of the cascade framework

Non-trivial proportions of individuals reported both successful attainment of steps in the cascade and a barrier to that step, for example, being motivated to use a prevention method and also reporting a barrier to motivation to use that prevention method. For those who reported using a prevention method and still report barriers to use, it can be interpreted that these people had sufficient capacity to use the prevention method that these barriers did not prevent them from using the prevention method of interest. Additionally, for the motivation bar, if people reported wanting to use a prevention method then any reported barriers to motivation are not sufficient to prevent motivation. However, the definition of access used in the cascade – knowing somewhere to access a prevention method – does not directly preclude the barriers to access in the cascade, such as affordability. The current definition of the access bar means individuals could be classified as attaining access (main bar) who still have barriers to access which prevent them actually accessing a prevention method. Further work to assess variation in overlaps by strength of views on the main bars and sub-bars, using Likert scale responses, could also help to improve definitions of the main and sub-bars.

Effective prevention method use is challenging to measure, particularly with male condoms and PrEP. In the absence of laboratory testing for the presence of PrEP drugs, which is impractical and expensive for large scale application of the prevention cascade, all measures of effective use of a prevention method rely on self-report which is subject to social desirability bias. The UNAIDS version of the HIV prevention cascade defines a focus population, similar to the Harare HPC priority population, and then reports reach/coverage uptake/use and correct/consistent use [29]. Taking into account consistent use is important when considering the ultimate goal of preventing acquisition of HIV but is difficult to measure using cross sectional survey data. Changing the definition of male condom use produced significantly different HIV prevention cascades for the same population. Ideally, effective use should be consistent use; however, this also needs to be balanced with the practicality of collecting data on consistency of prevention method use especially when adding questions to existing surveys and acknowledge that HIV risk can change rapidly such as through life course events. Current DHS questionnaire modules collect data on prevention method use at last sex and ever having used prevention methods, so the most feasible definition when using DHS data would be prevention method use at last sex.

Most of the explanatory barriers are clearly defined, however some barriers encompass several variables which could contribute to that barrier. This particularly affects ‘lack of skills’ which could include social skills to negotiate prevention method use but also practical skills to actually use or adhere to prevention. Combining these aspects limits the insight which could be gained from the framework about potential targets for interventions. More granular information could be obtained if these were separated. There may be some populations in which male condoms have been widely available for a long time. In these cases, it may be appropriate to assume that male condoms are always available, and nobody experiences the lack of availability barrier, particularly when the space or time available to ask questions on explanatory barriers are limited.

This HPC framework was designed to be used for individual and combination prevention. When addressing combination prevention, it is possible to present an overall view of motivation, access, and use of multiple prevention methods in the priority population. However, including information about the explanatory barriers to all prevention methods of interest is complicated and nuanced. There are multiple options for how the bars could be populated, such as barriers reported across all prevention methods, or barriers to use of the prevention method furthest along the prevention cascade. However, this does not provide as complete a picture of explanatory barriers as focusing on individual prevention methods. Garnett *et al*. suggested an alternative approach to populating combination prevention cascades in which an HPC is constructed for the most widely used prevention method first, then for the next most widely used method for those not using the first method [17]. Although this approach is less succinct, it may reveal information on sub-bars for each prevention method and could be done using the same data.

### Limitations of this validation exercise

This validation exercise was limited by use of self-reported cross-sectional data, which may lead to underestimation of the size of the priority population and overestimation of levels of prevention method use, however this should be somewhat mitigated by the use of informal confidential voting methods. Additionally, this HPC framework is designed to be populated using self-reported survey data so similar biases would be likely across other measurement.

Twenty-three percent of those eligible to complete the individual survey did not participate and 5% of participants did not consent to PITC or DBS collection which may introduce non-response bias to the population. However, similar limitations would be present using other population data, such as DHS survey data which does not contain HIV testing data. Due to the cross-sectional study design, it was not possible to address effective use of prevention method and the effect on HIV incidence, however multiple prevention methods have been proven to reduce HIV acquisition in other studies. When used correctly, condoms are highly effective in preventing transmission of HIV, giving an estimated reduction in transmission of 90–95% [46,47]. Voluntary medical male circumcision (VMMC) reduces HIV acquisition in men by between 53% and 60% [48–50]. The Zimbabwean national VMMC programme aims to reach 80% coverage of males aged 15–29 years by 2021 to reduce HIV incidence [51]. Clinical trials of oral pre-exposure prophylaxis (PrEP) have demonstrated its efficacy in preventing acquisition of HIV infection. Reported results vary, probably explained by differences in adherence [52–54] - with good adherence the effectiveness of oral PrEP was as high as 90% [55]. No validation was carried out for applying the cascade framework to use of female condoms, although the prevention cascade for these shows that motivation and use of these is very low.

Validation of the HPC framework applied to PrEP use was limited due to very small numbers of individuals reporting awareness of or use of PrEP, which only very recently became available in Zimbabwe [56]. Motivation to use and access to PrEP were collinear with PrEP use. Although this demonstrates that those who reported PrEP use were motivated to use and did have access to PrEP, further analysis in populations with higher levels of PrEP use is required to confirm these associations. The association of explanatory barriers with PrEP use could not be tested due to such small numbers reporting the outcome. However, results from this validation exercise (particularly objective 1) have shown that the Harare HPC framework provides valuable insights into gaps in the cascade (when measuring the core cascade) and subsequent barriers (from the extended cascade) when applied to both low use prevention methods (PrEP) and higher use methods (male condoms), and therefore can be applied to populations where both such cases exist.

Being motivated to have and reporting access to VMMC were associated with reduced odds of having VMMC, which is the opposite to what would be expected from the cascade framework. Ninety-five percent95% of people reporting VMMC responded that they were not motivated to get VMMC or did not know somewhere to access VMMC if they did want to access it. If this issue was not considered when populating the cascade, for VMMC or other prevention methods, it could appear as if more people were using a prevention method than were actually motivated to use it or had access to it. Other studies which have applied the HIV prevention cascade have not reported or explored this, however, most have used different endpoint measures of the cascade. Hensen et al used the HPC framework to identify gaps to increase coverage of VMMC services in Zambia [57], but the endpoint of the HPC framework was perceived service availability rather than actual uptake of VMMC. Some individuals reporting VMMC may have had VMMC when they were much younger, such as at school or as decided by their parents. Analysis of longitudinal data would be required to assess whether or not the men who have not yet taken up VMMC but do report motivation and access are more likely to do so compared to those who do not report motivation and access. Additionally, the reliance of self-reported data could have meant there was some misclassification of VMMC (for example including traditional circumcision as VMMC), which could mean VMMC was slightly overreported. However, study staff were trained and instructed in how to probe for full medical circumcision as opposed to traditional circumcision.

This validation exercise has only been tested in one population in Manicaland, east Zimbabwe. Further work using other populations, including alternative priority populations, would add to the credence of the HIV prevention cascade as a simple and effective way of understanding prevention method use, however there is a notable lack of data with which to be able to do this. There were some explanatory barriers which were not associated with reduced odds of prevention method use, such as access related barriers to condom use, possibly because condoms are so widely available and accessible. Additionally, some explanatory barriers showed statistically significant results but with a small effect size. However, both the strength of associations and the effect size may be significant in other populations due to population characteristics or sample size limiting detection of associations, and, therefore, the absence of associations within this population is not sufficient to warrant recommending removing these explanatory barriers from the cascade framework. The priority population chosen was a broad age range to maximise the sample size for analysis. Qualitative data suggest differences in attitudes to prevention method use in older versus younger people, highlighting the important of choosing relevant priority populations within appropriate age ranges [45]. However, as stated at the outset of this validation exercise, accepting or rejecting each hypothesised measured of validity should not be solely based on statistical outcomes, and lack of statistical association should not be taken as evidence to remove an aspect of the HPC framework. Consideration should be given to the underlying social cognitive theories which have informed the development of the Harare HPC framework. Recommendations for the population of and structure of the cascade framework

Based on this analysis, we recommend the following updates to the Harare. HIV prevention cascade:

1) It should be assumed that all individuals reporting prevention method use are motivated and have access to that prevention method
2) Where possible, the definition of the access bar should not include anyone who reports any barriers to access
3) Motivation and access should remain as currently ordered, with the exception of populations where access is close to 100%. In this case, more information may be gained from putting the access bar first to maximise the gaps in the cascade and aid identification of targets for interventions, but any reported access related barriers should still be assessed.
4) The lack of skills explanatory barrier should be split into 2 separate barriers: a lack of social skills and a lack of practical skills
5) Where possible, quantitative analysis should be combined with qualitative analysis to understand barriers to use of prevention methods, especially in populations where awareness and use of a particular prevention method is very low.

### Applying the HIV Prevention Cascade

Following considerable deliberation on the nature of the formulation of the HPC, good progress has been made on establishing the value of the HPC framework and the work done in this paper supports this. It has now been established, through this validation exercise, that it is possible to populate an HPC framework with data collected in a general population survey using questionnaire modules developed to facilitate this process. Through a combination of literature review, social and behavioural theory, and evaluation using real world data, a general consensus has been reached on what is important to include in a basic HIV prevention cascade and how such a framework can be used to support HIV prevention efforts, however consensus on the detailed formulation is still lacking.

Two predominant HIV prevention cascades have come to the forefront of work on HIV prevention monitoring: 1) the Harare formulation and 2) the UNAIDS formulation [18,29]. The Harare framework applied and further developed in this paper takes a user-centric perspective whereas the UNAIDS formulation takes a provider-centric perspective [18,29]. The perspective a cascade should take has been subject to much debate and each has its relative advantages and disadvantages [18,17,26,29].

Overall, it is possible to collect data as part of routine population surveys to populate the HIV prevention cascade. However, this required inclusion of specific questionnaire modules and this type of data may not be available without the specific questionnaires through which this can be collected. Using Demographic and Health Survey data, it could be possible to define some priority populations, populate the main bars of the cascade and thus generate a basic cascade used for high level monitoring and evaluation purposes. This could mimic the success of the treatment cascade in allowing identification of the gaps in the cascade, monitoring these over time and making comparison across and within countries. Where large gaps are found, this could be complemented with surveys to specifically explain these gaps and thus find appropriate targets for interventions.

Issues raised, and that remain, are not dissimilar to those raised in the adoption and implementation of the HIV treatment cascade. For example, parallel to the challenge of estimating a population at risk of acquiring HIV, challenges and some uncertainties exist in estimating the denominator for the HIV treatment cascade of PLHIV [17,58]. This is particularly relevant for treatment cascades which require estimation of the size of key populations [59–61]. Criticism of the HIV prevention cascade that individuals’ change in preventive behaviour is not captured [62] is also relevant to the treatment cascade which cannot assist in monitoring individuals who are lost from and subsequently reengaged in treatment services [63,64]. As yet, there are no published examples of countries or programmes adopting the UNAIDS version of the cascade using the definitions recommended as part of the Operational Guidance for Creating HIV Prevention Cascades. There are some emerging examples of the user-centric cascade approach being used by policy makers and programme managers. The national needle and syringe programme in Ukraine used the prevention cascade approach as a way of evaluating their programme using surveillance data, and, in Kenya, programme data on female sex workers were used to populate national and sub-national prevention cascades [30,65].

UNAIDS highlight that an HIV prevention cascade should be based on one data source in order to be truly comparable over time and specific to a population [29]. However, in practice, data used in cascades will not be perfect, such as in establishing a size estimate of the population at risk, i.e., the denominator of the whole cascade. It is highly likely that different data sources will feed into one cascade. Data sources, including population size estimates, routine programme data and behavioural surveys, could be expanded to include data to populate a cascade [29]. In order to reach the success of the HIV treatment cascade and fulfil its potential for cross-country comparison and advocacy, the HIV prevention cascade needs to be adopted into national monitoring and evaluation strategies. Indicators to measure at least one version of the HIV prevention cascade must be included in national surveys, such as the Demographic Health Survey (DHS) questionnaires. However, in order for this to happen there needs to be a consensus on which version(s) of the cascade the data will be collected for.

The UNAIDS version of the cascade is intended to be populated primarily using programme data that are already routinely available [29]. The main difference between this and the Harare cascade is that lack of motivation is not acknowledged within the UNAIDS cascade framework as an obstacle to use of prevention methods. Although gaps in programme coverage and use of prevention methods can be identified using the UNAIDS framework, when explaining these gaps and identifying relevant interventions, distinguishing motivation and demand-related factors from access-related explanatory factors is difficult. Additionally, cascades created using programme data do not provide information on people not accessing prevention services including prevention methods, and thus lack information on why people are not using prevention methods, except that they are not reached by the programme. As demonstrated in this analysis, motivation is strongly associated with prevention method use and reporting barriers to motivation is associated with reduced odds of using a prevention method, supporting the importance of addressing this step of the cascade to increasing prevention method use even in a scenario of very high access to primary prevention. Other efforts to populate this cascade framework, such as that looking at condom use in young women who sell sex in Zimbabwe, did not have data available on motivation to use condoms and so had to use a proxy measure of knowledge about condom efficacy [31].

#### Recommended use of the Harare HPC framework

For a standardised approach for comparing countries, there is a strong preference and benefit to using one version of the cascade framework in order to mimic the success of the treatment cascade and allow comparison across populations. As Auerbach *et al*. propose, a solution could be to use a 2-step process involving two cascades [26]. The first step would use the UNAIDS framework, leveraging commonly available programmatic data to populate the cascade and identify gaps. Once the gaps are identified, the Harare cascade can be used to further understand the demand side of prevention method use and reasons for gaps in the cascade using the hypothesised explanatory sub-bars. Given the demonstrated importance of motivation within the cascade framework, adding a question on motivation wherever possible, such as to routine surveys, would provide valuable information on the gaps in individual level motivation to use primary prevention methods. The latest WHO Strategic Information guidelines on HIV prevention emphasise demand-led referrals to primary prevention services [66]. Monitoring of this could mean that data on whether or not individuals seen in routine programmes want (are motivated) to use a prevention method would need to be collected in programme records, thus making is possible to measure the Harare HPC using programme data. However, these records would still only capture data on people who access or are reached by the programme, which would be reflected in the denominator of the cascade.

Much commentary around the potential utility of HIV prevention cascades mentions that, to mimic the success of the treatment cascade, targets need to be attached to bars of the prevention cascade akin to the’90-90-90’ targets of the treatment cascade [18,26,67]. However no global targets for HIV prevention that could be mapped to the prevention cascade had been set until recently [26]. Targets set out in the 2025 UNAIDS roadmap could be linked to comparable targets for the HIV prevention cascade bars [68]. A target of “95% of people at risk of HIV use appropriate, prioritised, effective combination prevention” could be attached to the end point of the HPC: effective use [68]. Prevention targets for key populations set for use of individual prevention methods provide clear targets for end-points of cascades for specific priority populations, e.g., “90% of all sex workers reporting condom/lubricant use at last sex with a client or nonregular partner” [68]. Broader targets exist as well such as for condom use by young people and adults aged 15–49 years and not taking PrEP [68]. The milestone of “Strengthen subnational monitoring and evaluation systems, including non-health data, and put the subnational scorecard system into operation” could implement the HIV prevention cascade framework as a standardised way of monitoring and evaluation prevention programmes. This analysis demonstrates that it is possible to apply the HPC framework to a priority population identified through a general population survey. Future work to promote the HPC must encourage the adoption of key questions into national surveillance and demonstrate how the framework can provide insight of how prevention efforts compare to these UNAIDS targets, as well as identifying targets for interventions necessary to meet these targets.

Schaefer *et al.* note that the Harare proposed version of the cascade could be applied to mathematical modelling to predict the infections averted by current HIV prevention use and predict the impact on HIV incidence of reducing barriers within the cascade [18], as also suggested by Auerbach *et al.* [26], and demonstrated by Pickles *et al* using data from this study to parameterise the model using this version of the cascade framework [69].

### Final recommended formulation of the HIV Prevention Cascade

The final proposed formulation of the HIV prevention cascade is illustrated for men’s use of male condoms in Fig 10. Depending on the availability of data, the cascade can be populated as the full cascade including explanatory barriers to each step, or just as the main bars of the cascade whereby the difference in the bars indicates the gaps where people are being lost from the cascade. In the latter case, once these gaps are identified, further work can be done to establish the cause of these gaps.

### Recommended steps to populating the HIV Prevention Cascade

The following steps should be taken to populate this HIV prevention cascade:

1) **Priority population:** Establish the priority population as a population that would benefit from using HIV prevention according to the population or research question of interest
2) **Main bars:**
  a. **Use:** Calculate the number within the priority population using the HIV prevention method of interest according to the chosen definition of effective use. Suggested definitions are listed in S5 Table.
  b. **Motivation:** Calculate the number within the priority population who are motivated to use the HIV prevention method of interest according to the chosen definition of motivation. Suggested definitions are listed in S5 Table. Recode all individuals who are reporting use but not motivation to be motivated.
  c. **Access:** Calculate the number within the priority population who are motivated to use and report access to the HIV prevention method of interest according to the chosen definition of access. Suggested definitions are listed in S5 Table. Recode all individuals who are reporting use but not access to have access. Where data is available, recode all individuals reporting motivation and at least one barrier to access as not having access.
  d. **Calculate motivation, access, and use as proportions:** Each main bar of the cascade is presented as a proportion with the calculations:

$$Proportion\;motivated=\;\frac{\begin{array}{c} number\;of\;individuals\;reporting\;to\;be\;motivated\;\\ to\;use\;a\;prevention\;method \end{array}}{number\;of\;individuals\;in\;priority\;population}$$

$$Proportion\;with\;access=\;\frac{\begin{array}{c} number\;of\;individuals\;reporting\;to\;be\;motivated\;to\;use\;\\ and\;have\;access\;to\;a\;prevention\;method \end{array}}{number\;of\;individuals\;in\;priority\;population}$$

$$Proportion\;effectively\;using=\;\frac{\begin{array}{c} number\;of\;individuals\;reporting\;to\;be\;motivated\;to\;use,\\ have\;access\;to\;and\;be\;effectively\;\\ using\;a\;prevention\;method \end{array}}{number\;of\;individuals\;in\;priority\;population}$$

95% confidence intervals for each main bar proportion can be calculated and displayed around the main bars of the HPC framework.

3) **Explanatory sub bars:** Where data are available explanatory sub bars for each step can be populated using suggested definitions in S6 Table. The explanatory sub bars should be limited to those falling within the gaps between each of the main bars in the cascade.

Motivation related sub-bars should only be experienced by those who are in the priority population but unmotivated. Access related sub-bars should only be experienced by those in the priority population who are motivated but do not report access. Effective use sub-bars should only be experienced by those in the priority population who are motivated and have access but do not report using the prevention method of interest.

4) **Combination prevention:** Where data are available, the measures of individual prevention method motivation, access and use can be combined to produce combination prevention cascades. Depending on the population of interest and research questions, criteria for combination motivation, access or use can either be:
  a. **Using at least one prevention method –** bars for combination use should be created where individual meeting the criteria for the respective bar for at least one prevention method fall within that bar
  b. **Using multiple prevention methods at the same time -** bars for combination use should be created where individuals meeting the criteria for the respective bar for at all prevention methods of interest fall within that bar

Main bars only should be populated for combination prevention cascades. If gaps are identified from this analysis, the cascades should be split into individual prevention methods and at this point explanatory sub bars should be populated to understand the barriers relevant to each prevention method. At this point, common barriers across prevention methods within the priority population could be identified.

5) **Comparison to national/international targets –** calculate the percentage of the priority population who report motivation, the percentage of those motivated who report access and the percentage of those motivated and with access who report effect use. Compare these with the 90-90-90 equivalent targets where available, such as those set out in the UNAIDS HIV Prevention 2025 Road Map [68,70].

## Conclusions

Overall, it was possible to measure the HPC for individual and combination prevention using the questionnaires module designed for the Manicaland pilot survey. Valuable insights into levels, of gaps and barriers to prevention method use were gained when prevention method use was both high and low. It has now been established, through our work, that it is possible to populate an HPC framework with data collected in a general population survey using questionnaire modules developed to facilitate this process. This would be strengthened through repeating this exercise in other populations and epidemiological context when data becomes available. Through a combination of literature review, social and behavioural theory, and evaluation using real world data, a consensus has been reached on what is important to include in a basic HIV prevention cascade and how such a framework can be used to support HIV prevention efforts. If sufficiently evaluated and promoted, the HIV prevention cascade can contribute to improvements in HIV prevention service provision and help to bring about necessary reductions in HIV incidence to end the HIV/AIDS epidemic as a global public health threat.

## Supporting information

S1 Table Male condom cascade main bar and sub bar definitions. (DOCX)

S2 Table PrEP cascade main bar and sub bar definitions. (DOCX)

S3 Table VMMC cascade main bar and sub bar definitions. (DOCX)

S4 Table Sociodemographic characteristics of participants completing individual questionnaire with a negative HIV result. (DOCX)

S5 Table Proposed revised definitions of each main bar of the HIV prevention cascade. (DOCX)

S6 Table Proposed revised definitions of each explanatory sub-bar in the HIV prevention cascade. (DOCX)

S1 Checklist Inclusivity in global research. (DOCX)
