# Supplementary material for: Appraising the HIV prevention cascade methodology to improve HIV prevention targets: Lessons learned from a general population pilot study in east Zimbabwe
Source: PLOS Glob Public Health. 2026 Feb 10;6(2):e0005336. doi: 10.1371/journal.pgph.0005336 (PMC12890129; doi:10.1371/journal.pgph.0005336)
Supplement: S4 Table — (DOCX) [file pgph.0005336.s004.docx]

S4 Table - Sociodemographic characteristics of participants completing individual questionnaire with a negative HIV result

|  |  | Started sex | | |  | Priority population | | |
| --- | --- | --- | --- | --- | --- | --- | --- | --- |
|  |  | Female  15-54 years  N = 3173 |  | Male  15-54 years  N = 2050 |  | Female  15-54 years  N = 575 |  | Male  15-54 years  N = 444 |
|  |  | % (95% CI) |  | % (95% CI) |  | % (95% CI) |  | % (95% CI) |
| Site type |  |  |  |  |  |  |  |  |
| Urban |  | 18.2 (16.8-19.5) |  | 14.2 (12.7-15.8) |  | 14.2 (11.2-17.8) |  | 16.0 (14.0-20.2) |
| Peri-urban |  | 27.8 (26.2-29.4) |  | 23.1 (21.3-24.9) |  | 24.5 (20.8-28.8) |  | 21.0 (17.9-24.6) |
| Estates |  | 23.9 (22.4-25.4) |  | 34.8 (32.7-36.9) |  | 27.5 (23.5-31.8) |  | 39.7 (35.7-43.7) |
| Rural |  | 30.2 (28.6-31.8) |  | 28.0 (26.0-29.9) |  | 33.8 (29.5-38.3) |  | 22.4 (19.2-26.0) |
|  |  |  |  |  |  |  |  |  |
| Education |  |  |  |  |  |  |  |  |
| None/primary |  | 18.6 (17.3-20.0) |  | 10.6 (9.3-12.0) |  | 17.8 (14.5-21.6) |  | 9.4 (7.3-12.1) |
| Secondary/higher |  | 81.4 (80.0-82.7) |  | 89.4 (88.0-90.7) |  | 82.2 (78.4-85.5) |  | 90.6 (87.9-92.7) |
|  |  |  |  |  |  |  |  |  |
| Marital status |  |  |  |  |  |  |  |  |
| Never married |  | 7.8 (7.0-8.8) |  | 24.0 (22.2-25.9) |  | 24.1 (20.3-28.3) |  | 38.6 (34.7-42.7) |
| Currently married |  | 78.7 (77.2-80.1) |  | 71.2 (69.1-73.0) |  | 44.8 (40.2-49.5) |  | 52.3 (48.3-56.4) |
| Divorced/separated |  | 10.4 (9.4-11.5) |  | 4.6 (3.8-5.6) |  | 27.7 (23.7-32.1) |  | 9.0 (7.0-11.7) |
| Widowed |  | 3.1 (2.5-3.8) |  | 0.3 (0.1-0.7) |  | 3.4 (2.0-5.5) |  | 0.0 |
| Socioeconomic status |  |  |  |  |  |  |  |  |
| Poorest |  | 8.7 (7.8-9.8) |  | 9.1 (7.9-10.4) |  | 9.7 (7.3-12.8) |  | 9.0 (7.0-11.7) |
| 2nd poorest |  | 42.1 (40.4-43.8) |  | 47.3 (45.1-49.4) |  | 47.3 (42.7-52.0) |  | 46.3 (42.2-50.4) |
| 3rd poorest |  | 24.5 (23.0-26.0) |  | 23.8 (22.0-25.7) |  | 23.0 (19.3-27.1) |  | 21.6 (18.4-25.1) |
| 4th poorest |  | 23.2 (21.8-24.7) |  | 18.7 (17.1-20.4) |  | 18.9 (15.5-22.8) |  | 21.7 (18.6-25.3) |
| Least poor |  | 1.5 (1.1-1.9) |  | 1.2 (0.8-1.7) |  | 1.1 (0.5-2.7) |  | 1.4 (0.7-2.8) |
